# αII-Spectrin Regulates Invadosome Stability and Extracellular Matrix Degradation

**DOI:** 10.1371/journal.pone.0120781

**Published:** 2015-04-01

**Authors:** Aurélie Ponceau, Corinne Albigès-Rizo, Yves Colin-Aronovicz, Olivier Destaing, Marie Christine Lecomte

**Affiliations:** 1 Institut National de la Transfusion Sanguine, INSERM UMR-S 665, Paris, France, Université Paris 7/Denis Diderot, Paris, France; 2 Institut Albert Bonniot, Université Joseph Fourier, Centre National de la Recherche Scientifique, Institut National de la Santé et de la Recherche Médicale-Université Joseph Fourier U823 Site Santé, Grenoble, France; King's College London, UNITED KINGDOM

## Abstract

Invadosomes are actin-rich adhesion structures involved in tissue invasion and extracellular matrix (ECM) remodelling. αII-Spectrin, an ubiquitous scaffolding component of the membrane skeleton and a partner of actin regulators (ABI1, VASP and WASL), accumulates highly and specifically in the invadosomes of multiple cell types, such as mouse embryonic fibroblasts (MEFs) expressing SrcY527F, the constitutively active form of Src or activated HMEC-1 endothelial cells. FRAP and live-imaging analysis revealed that αII-spectrin is a highly dynamic component of invadosomes as actin present in the structures core. Knockdown of αII-spectrin expression destabilizes invadosomes and reduces the ability of the remaining invadosomes to digest the ECM and to promote invasion. The ECM degradation defect observed in spectrin-depleted-cells is associated with highly dynamic and unstable invadosome rings. Moreover, FRAP measurement showed the specific involvement of αII-spectrin in the regulation of the mobile/immobile β3-integrin ratio in invadosomes. Our findings suggest that spectrin could regulate invadosome function and maturation by modulating integrin mobility in the membrane, allowing the normal processes of adhesion, invasion and matrix degradation. Altogether, these data highlight a new function for spectrins in the stability of invadosomes and the coupling between actin regulation and ECM degradation.

## Introduction

Identified at the inner surface of the erythrocyte membrane, spectrins are the central components of a universal and complex spectrin-actin scaffold, called the spectrin-based skeleton [1]. Spectrins, as flexible large/long tetramers (200 nm length) composed of α and β subunits that associate side by side then head to head constitute the filaments of this network. In mammals, spectrins are encoded by seven genes, including two genes for the α-spectrin subunits (αI and αII), and five genes for the β-spectrin subunits (βI to βV). The αβ heterodimers display distinct tissue-specific cellular and subcellular patterns of expression: red-blood-cell spectrin consists of exclusive association of αI and βI subunits whereas αII and βII to βV combinations are present in non-erythroid cells. Thus, the spectrin network is attached to diverse cellular membranes through interaction with various transmembrane proteins, either directly or involving adaptor proteins such as ankyrins and proteins 4.1 (belonging to the FERM family).

Most spectrin functions have been clearly defined from numerous studies on red cells, particularly those on hereditary hemolytic anemia, which have clearly established the importance of spectrins for supporting cell shape and plasma membrane stability [2, 3]. In non-erythroid cells, the functions of spectrins are less clear; however, they have been implicated in the establishment and the maintenance of specialized membrane domains. Loss of spectrins is associated with mislocation of membrane protein such as the TRCP4 channel, voltage-gated channels (βIV-spectrin), the glutamate transporter EAAT4 (βIII-spectrin) or the Na/K-ATPase (βH and βII-spectrin) [4, 5]. Spectrins can also bind and regulate adhesion molecules such as Lu-BCAM and L1-CAM [6, 7]. Using siRNA and genetic approaches, recent data demonstrated a critical role of αII-spectrin in cell adhesion, actin organization, growth and proliferation of primary and transformed cells [8]. Spectrins have also been described in numerous cellular contacts such as intercellular tight junctions (through interaction with ZO-1), gap junctions (through interaction with connexin 43) and adherens junctions (through direct interaction with α-catenin and E-cadherin/β-catenin/αII-spectrin complex) [9–15].

The link with integrin-based adhesions is less clear. Despite the fact that spectrins participate in the organization of focal adhesions, αII-spectrin has never been clearly localized to these structures [16, 17]. However, αII-spectrin has recently been localized to other adhesion structures such as podosomes [18]. Podosomes and invadopodia are adhesive mechanosensory modules composed of a dense F-actin core surrounded by a ring of adhesion molecules. As the distinction between podosomes and invadopodia is still a matter of debate, these structures will be grouped herein under the term invadosomes [19–22]. The invadosome units can auto-organize into metastructures, forming dynamic rings, which expand in diameter, often fuse with each other and disappear due to continuous remodeling. This is resulting from the coordinated assembly of new invadosomes at their outer rim and disassembly of older ones at the inner rim [23–25].

Invadosomes can be induced either by expressing a constitutive active mutant of Src, SrcY527F, or by activation of protein kinase C isoforms (PKCs) through phorbol ester or growth factor treatment such as EGF or TGF-β [26–29].

Invadosomes are the sites of metalloprotease activities necessary for their extracellular matrix (ECM) degradation activity and are implicated in invasion processes [30–33]. The invadosome activities of actin remodelling and ECM degradation can be uncoupled under specific conditions where the activation of β1-integrins or the level of Tks4 phosphorylation is perturbed [34, 35].

To better understand the role of αII-spectrin in cell adhesion and motility, we have determined the localization and expression of αII-spectrin and its potential β-spectrin partners in invadosomes from different cell types (HMEC-1 and MEFs expressing an active mutant of Src). Additionally, we further determined the involvement of αII-spectrin in invadosome formation and functions, using several approaches such as knockdown of αII-spectrin expression and live imaging.

## Materials and Methods

### Cell culture

The Human Microvascular Endothelial cell line, HMEC-1 (ATCC, CRL-10636) was grown in MCDB131 (Gibco) containing 15% FCS (FCS PAN Biotech GmbH), 2 mM L-glutamine, 1μg dexamethasone (D8893, Sigma) and 100 ng EGF (Invitrogen). The MEF Src Y527F cell line (Mouse Embryonic Fibroblast transformed by a constitutively active form of the kinase Src, as described in [35, 36], was cultured in DMEM GlutaMAX-I (Gibco) containing pyruvate, glucose (4.5g/L) and supplemented with 10% FCS (FCS PAN Biotech GmbH). An antibiotic/antimicotic mixture (penicillin, 10,000 unit/ml and streptomycin 10 ng/ml, Invitrogen) was added in all culture medium.The HMEC-1 cells were grown on plastic coated with 0.2% gelatin (Sigma Aldrich, France) and the MEF v-SRC Y527F on plasticat 37°C in water-saturated atmosphere with 5% CO2.

### Transfection and plasmids

Cells were transfected with short hairpin RNA plasmids (shRNA, SA Biosciences) expressing GFP and a non-targeting sequence used as control (non-relevant shRNA, named Nr-shRNA) or a sequence targeting the αII-spectrin gene, (αII-spectrin shRNA named Sp-shRNA) using JET PEI reagent (Ozyme-Polyplus), according to the manufacturer’s instructions. Transfection efficiency was determined by flow cytometry (GFP expression) 24 hr after transfection. Four Sp-shRNA plasmids were tested: for mice cells referred as 1m, 2m, 3m and 4m and for human cells referred as 1 h, 2 h, 3 h and 4 h).Depletion efficiency of αII-spectrin expression was estimated by western blot at 24, 48, 72 and 96 hr after transfection. Other plasmids were also used to transfect MEF v-Src Y527F cells: recombinant full-length of αII-spectrin fused to GFP from pCep4 plasmid (pCep4 GFP αII-spectrin, generous gift from Dr Gaël NICOLAS), and plasmids expressing the LifeAct peptide fused with fluoro-Ruby (Ruby-LifeAct, a red marker visualising F-actin in living cells), β3-integrin tagged with RFP (clone 285, β3-Integrin RFP), actin tagged with RFP(RFP-actin), cortactin tagged with RFP (clone 145, RFP-Cortactin), or paxillin tagged with RFP (called RFP-paxillin in experiments), these sixth last allowing detecting invadosome structures.

### Reagents and antibodies

Phorbol-12-myristate-13-acetate (PMA, 50ng/ml final concentration) and recombinant EGF (5 ng/ml final concentration) were obtained from Sigma Aldrich (France). Recombinant TGF-β1 (used at 5 ng/ml) was purchased from Invitrogen Life Technology (France).

Polyclonal antibodies directed against αII-spectrin were obtained after immunization of guinea pigs (Eurogentec) using the recombinant 6xHis tagged peptide encompassing the α8-α11 repeats, including the SH3 domain from human αII-spectrin. Sera collected from two guinea pigs (S7 and S8) were used for immunostaining detection of αII-spectrin. A monoclonal antibody directed against αII-spectrin (clone AA6, from Millipore) was also used to detect protein expression. The different β-spectrins were detected by rabbit polyclonal anti-βI-spectrin produced after immunization with the purified βI-spectrin chain, mouse monoclonal anti-βII-spectrin obtained from BD Biosciences (clone 42), rabbit polyclonal anti-βIII-spectrin (clone H70), goat polyclonal anti- βIV-spectrin (clone C-16) and goat polyclonal anti-βV-spectrin (clone C13) from Santa Cruz Biotechnology. Rabbit polyclonal antibodies against cortactin, phospho-FAK, paxillin (clone 349), ABI-1, MMP2, MMP9, and MMP14 were obtained from Abcam; phopho-cortactin [pY421] from Invitrogen; VASP and WASL from Sigma-Aldrich, Lamin A/C from Santa Cruz Biotechnology. Monoclonal antibodies directed against Src (clone GD11) and PKC (clone M110) were obtained from Upstate Biotechnology and Abcam, respectively. Monoclonal antibodies directed against β1- and β3-integrin were from Abcam and 9EG7 antibody (anti activated β1-integrin) was from BD Pharmingen. Alexa Fluor-488 or Alexa Fluor- 568-labelled secondary anti-IgG antibodies were purchased from Molecular Probes. Alexa Fluor-568or -488-phalloidin (Molecular Probes, France) were used to label F-actin.

### Western blot analyses

After two washes with prewarmed Dulbecco’s PBS (D-PBS Gibco), cells were directly lysed on plates in PBS containing 1% SDS and an anti-protease cocktail (Sigma Aldrich, France). Protein concentrations were estimated by a colorimetric assay using the BCA method (microAssay Uptima, Interchim, France), with BSA as standard. SDS sample lysates were analyzed on a 4–12% SDS-polyacrylamide gel (InVitrogen, France), followed by western blotting using the appropriate antibodies. Briefly,proteins were transferred onto nitrocellulose membranes (0.45 μm) (Protan, Schleicher & Schuell, France) using a Tris-glycine buffer. After saturation with PBS buffer, pH, 7.5 containing 5% non-fat-milk, and 0.05% Tween 20, the membranes were probed with the primary antibodies overnight at 4°C. After extensive washing, blots were incubated for 1 hr at room temperature with secondary antibodies conjugated with horseradish peroxidase (Jackson immunoresearch, France). Immune complexes were detected using the Supersignal West Pico chemiluminescence substrate (Pierce, ThermoScientific, France). Chemiluminescence was quantified using the Quantity One 1-D Analysis software (Bio-Rad, France) after acquisition with the Molecular Imager Gel Doc (Bio-Rad, France).

### RT-PCR analyses

RNA was extracted from cells with the RNA Cleanup RNeasyMini kit using Spin technology (Qiagen, France), according to the protocol suggested by the supplier. Reverse transcription (RT) was performed from 1 μg of RNA (Abgene kit, Abgene, France) for 10 min at room temperature and then 30 min at 42°C. Primers for PCR (Eurofins, France, and gift from Dr Aziz El Amraoui, Institut Pasteur) were the following:

First PCR:

αII-spectrin: 5’**TCGGCTTTCAATAGCTG** 3’ (F); **5’CCAGGTTGTGCTGCAT 3’**(R)

βI-spectrin: **5’AGTTCTCGAGGGATGC 3’**(F); **5’GTGCTGATGGTGACAG 3’**(R)

βII-spectrin: **5’TATGCAGGGGACAAGG 3’**(F); **5’TCTCTCATCCCAGGTC 3’**(R)

βIII-spectrin: **5’ AGAGAGGCAGACCCT 3’**(F); **5’GTCCCTGGCAGTTTTC 3’**(R)

βIV-spectrin: **5’ AGCCTGTACTGTGTGC 3’**(F); **5’AAGAATGGAGCCCCAG 3’**(R)

βV-spectrin: **5’ AGATCCATAGCCACAAG 3’**(F); **5’GCCTGCTAGATCCTGT 3’**(R)

Nested PCR:

αII-spectrin: **5’ATCCCTCAGCTCTGCA 3’**(F); **5’TCCAGGATGAGGGCTT 3’**(R)

βI-spectrin: **5’AAGTCCACAGCCAGCT 3’**(F); **5’GGATCTGAAGGAGGCT 3’**(R)

βII-spectrin: **5’GCTGGTAGACACAGGA 3’**(F); **5’AAGCAGCCAAGCCTCT 3’**(R)

βIII-spectrin: **5’GGAACAGATGGAAGGG 3’**(F); **5’CACCACTGGCTCTTCT 3’**(R)

βIV-spectrin: **5’TGCACAAAGCCACCAG 3’**(F); **5’AGTCATTCCGGCCCTT 3’**(R)

βV-spectrin: **5’CTTAGAGACAGAGGCC 3’**(F); **5’TGCATGGGCCTCTCT 3’**(R)

The first PCR was done in 25 μl contained 2.5 μl of RT products, 12.5 μl of "Power SYBR Green PCR Master Mix" (Applied Biosystems), 2μl of oligonucleotides (10μM) and 8.5 μl of water (30 cycles; 94°C 45''; 58°C 45''; and 72°C 45''). Nested-PCR contained 4μl of first PCR and was performed for 40 cycles. The PCR products were loaded on a 2% agarose gel and separated at 100V. Amplified fragments were approximately 600 bp for the first PCR, and 300 bp for the second PCR.

### Immunofluorescence staining

Cells were gently washed in prewarmed D-PBS, fixed in 4% paraformaldehyde for 30 min at 37°C, then permeabilized with 0.5% Triton X-100. Preparations were saturated 30 min with Image-iT Signal Enhancer (InVitrogen Life Technology, France). Primary and secondary antibodies were diluted in background reducingbuffer (Dako antibody reagent with background reducer, InVitrogen Life Technology, France). Immuno-labelled cells were mounted in a ProLong Antifade Gold solution (InVitrogen Life Technology, France) before analyses.

### Live cell analysis

MEF v-Src Y527F cells were co-transfected with Ruby-LifeAct, and either Nr-shRNA or Sp-shRNA. Global cell dynamics, migration properties and invadosome dynamics were analysed 96 hr after transfection. Cells were plated in IQ4 slides (Biovalley) at a density of 100,000 cells/ml and incubated at 37°C in 5% CO_2_. The different parameters were registered in the Biostation system (Biostation IM, Nikon) during 12 hr, with cycles of 3 min each. Ten fields were analyzedfor each experimental condition. For Total Internal Reflection Fluorescence microscopy (TIRFM) and Fluorescence Recovery After Photobleaching (FRAP), MEF cells v-Src Y527F were transfected with pCEP4 αII-spectrin GFP plasmids, and also with plasmid Ruby-LifeAct or shRNA expressing RFP-Paxillin, RFP-Cortactin or the β3-integrin. Transfected cells were seeded at a sub-confluent density on borosilicate Lab-Tek slides with two chambers (Thermo Scientific, France) in a CO_2_-independent medium (InVitrogen, France). Localization of αII-spectrin and invadosome components (actin, paxillin, cortactin and β3-integrin) was selectively visualized at the plasma membrane in living cells using a TIRF microscope optimized for TIRF analysis. Mobility of αII-spectrin and other invadosome components were determined by FRAP technology.

### Microscopy and image analysis

Images were collected using an inverted laser scanning confocal microscope (Eclipse TE300 confocal system, Nikon), equipped with either 60X (0.17 WD 0.13) or 100X (0.17 WD0.20) oil-immersion-objectives. Image sets to be compared were acquired with EZ-C1 confocal software acquisition (IDS format) at pinhole S during the same session and using the same acquisition settings. Background intensities were equalized to a similar level in all images. Colors were obtained by selective laser excitation at 488 nm (green) and 543 nm (red). Phase-contrast was performed using an inverted Eclipse Ti microscope (Nikon) equipped with a 10X objective (0.17 WD 0.16), and images were acquired with a camera (Tiff format). For Total internal reflection fluorescence microscopy experiments, an Axiovert 200M microscope equipped with a 63X (NA 1.4) plan apochromat objective and triple-filter set 25HE (excitation, TBP 405 + 495 + 575 [HE]; beam splitter, TFT 435 + 510 + 600 [HE]; emission, TBP 460 + 530 + 625 [HE]) (Carl Zeiss Microimaging) was used for illumination. For fluorescence recovery after photobleaching experiments, an LSM510 ConfoCor microscope equipped with a 40X (NA 1.2) with a plan apochromatic objective (Carl Zeiss Microimaging) was used.

Fluorescent images were processed with EZ-C1 viewer then Adobe Photoshop CS5 and phase-contrast images with Adobe Photoshop CS5. TIRF images were analyzed using Metamorph software and Image J. Video acquisition were assembled with Image J software and BioStation IM. Quantification of cells showing podosomes was done by counting around 300 cells for each coverslip. Determination of invadosome size and thickness was evaluated by analyzing 500 rosettes per conditions using the ImageJ software. FRAP data were analyzed with the FRAP module of the Zen blue software (Carl Zeiss Microimaging). To summarize, FRAP data were normalized by measuring the background level and compared to fluorescence intensity of invadosomes that were not photobleached. The fluorescence recovery after bleaching time (3.2 s) was observed primarily in actin spots that persisted throughout the entire recovery time. Through the use of a single exponential fitting approach, the average characteristic time of recovery and immobile fraction were determined from at least 32 cells from 3 independent experiments.

Quantification of cell degrading FITC-labelled gelatin was performed for at least 10 fields (10X objective) for each coverslip. Invasion was quantified by counting on 10 fields the number of invading cells for each well (10X objective).

### Static cell adhesion assays

Adhesion assays were performed 96 hr after transfection with either Nr- or Sp-shRNA. After two washes in D-PBS, cells were detached using a trypsin-EDTA solution, and suspended 30 min in complete culture medium before plating in triplicate on 12 well-plates (2 x 10^6^ cells per well). Adhesion was analysed 0, 10, 20, 30, 60 and 120 min after plating. At the indicated times, cells were directly fixed in the wells with paraformaldehyde (4%) for 15 min at room temperature. After washing with D-PBS, the remaining adherent cells were visualized using an Eclipse Ti microscope (Nikon). Ten images were acquired for each sample, and adherent cells were counted using the Image-Pro Plus software. Results were expressed as the mean percentages of adherent cells per field.

### Matrix degradation assay on FITC-labelled gelatin

MEF v-Src Y527F cells were co-transfected with Ruby-LifeAct, and either with GFP-irrelevant shRNA or GFP-shRNA targeting αII-spectrin, then seeded for 16 hr on FITC-gelatin coated coverslips, as described previously [37–39]. Co-localization between dark areas and invadosome rosettes was visualized. Images were acquired every 3 min using the Biostation system (Biostation IM, Nikon).

### Expression of matrix metalloprotease proteins by zymography

MEF v-Src cells seeded at a density of 200 000 cells/ml were incubated overnight in serum-free medium. Supernatants were collected and immediately conserved at -80°C. Samples (20 μl supernatant and 20 μl 2X NovexTris-Glycine SDS sample buffer) were loaded on Zymogram gels containing 10% NOVEX 0.1% gelatin (InVitrogen Life Technology, France) and separated with 1X Tris-Glycine-SDS running buffer at 125 V for 90 min. Proteins were renatured in a Zymogram renaturing buffer (InVitrogen Life Technology, France) at room temperature for 30 min and developed in a Zymogram Developing buffer (InVitrogen Life Technology). Protein labeling was performed using Simply Blue SafeStain Coomassie G250 (InVitrogen Life Technology, France) and revealed after 1 hr washing with D-PBS. Gels were placed in a protective plastic film and scanned at a resolution of 300 dpi or more. The images were saved in a TIFF format and analyzed using Image J software.

### Invasion assay in Matrigel Invasion Chamber

Invasion chambers coated with Matrigel (BD Biosciences) were thawed at room temperature, then placed in a 24-well plate and rehydrated with serum-free medium at 37°C and 5% CO2. After 2 hr incubation, the medium was carefully removed without disturbing the Matrigel layer. MEF v-Src Y527F (5x10^4^ cells in 500 μl serum-free medium) were plated in the inserts and the lower chamber was filled with complete medium (1 ml). During 24 hr, serum deprivation causes attraction of cells to the lower chamber, resulting in transmigration of cells through the filter. Non-migrating cells were scrapped from the upper side of the insert filters and then the inserts were rinsed by D-PBS. Migrating cells present on the lower side of the inserts were labeled with a toluidine blue solution (basic dye with metachromatic properties). Images were collected using phase-contrast microscopy, and ten images were registered and analyzed per condition. Results were expressed as the mean percentages of migrating cells per field.

### Statistical analysis

Each experiment was performed at least in triplicate, and values represent the means of at least three independent experiments. Significance was determined using the Student’s t-test, and a 95% confidence interval was set a priori as the desired level of statistical significance.

## Results

### αII-Spectrin is present in the invadosomes of different cell systems

**Fig 1:**
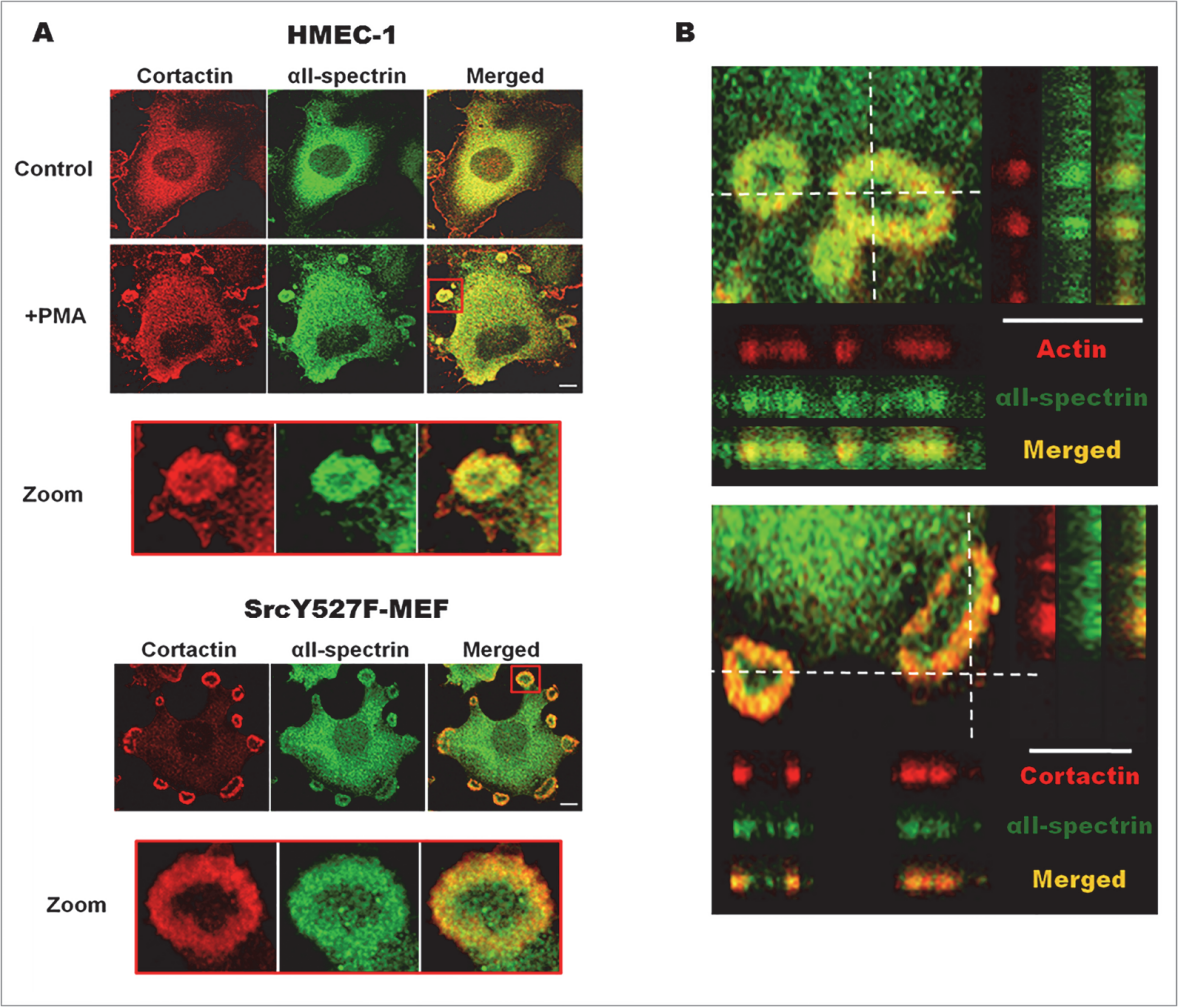
αII-Spectrin is localized in different invadosomes. (A) αII-Spectrin localization in PMA-treated HMEC-1 cells and SrcY527F-MEF cells. Starved HMEC-1 cells were treated for 1hr with PMA (50 ng/ml) in order to induce characteristic invadosome rings (zoom red square). Then, endogenous cortactin and αII-spectrin were stained and αII-spectrin relocalization after invadosome induction visualized. The presence of endogenous αII-spectrin was also seen in invadosome rings from SrcY527-MEF cells. (B) 3D localization of αII-spectrin in invadosomes. Confocal Z-stacks were obtained in SrcY527F-MEF cells stained for cortactin or F-actin. αII-Spectrin dashed lines represent the projected areas (Y section is projected on the right and X section on the bottom of the image), showing preferential αII-spectrin localization at the basis of the invadosome ring. Scale bar: 5μm.

Previous work localized αII-spectrin in podosome-like structures of non-invasive carcinoma cells. To expand this observation, we determined the localization of endogenous αII-spectrin in different invadosome models such as in human endothelial cells (HMEC-1 cell line), primary human endothelial cells (HUVEC) after different treatments (PMA or EGF) leading to invadosome formation, and in mouse embryonic fibroblasts expressing a constitutively activated mutant of the non-receptor tyrosine kinase Src (SrcY527F). HMEC-1 cells were treated for one hour with PMA (50 ng/ml), a well-known agent that induces invadosome assembly via PKC activation, and approximately 15% of cells were able to assemble invadosome structure-like rings as revealed by cortactin staining (red, Fig. 1A). In non-treated HMEC-1, αII-spectrin staining (green, Fig. 1A) was mostly localized in the cytoplasm and in the membrane at the edge of some cell protrusions. PMA treatment induced the recruitment of αII-spectrin in invadosome rings and localized nicely with cortactin. In a similar way, αII-spectrin relocalized into invadosomes of HMEC-1 cells that were induced by either EGF (5 ng/ml, S1A Fig.) or TGFβ (5 ng/ml, S1B Fig.). The presence of αII-spectrin in invadosomes seems to be a general feature as αII-spectrin was also found in invadosomes of MEF cells expressing the constitutively active mutant of Src, SrcY527F (Fig. 1A). Although invadosome cores are difficult to localize in these models, αII-spectrin did not co-localize with cortactin hot spots that are present in invadosome rings. Instead, αII-spectrin appeared to localize to the actin cloud surrounding the actin core (zoom Fig. 1A and B). As invadosomes are dome-shaped structures extending orthogonally from the basement membrane, αII-spectrin was localized in3D reconstructed invadosome. Based on orthogonal projections of invadosome rings in SrcY527F-MEFs, αII-spectrin was mostly present at the basis of the invadosomes. This confirms αII-spectrin localization in the actin cloud rather than in the core, and suggests a potential function for this protein at the interface between the membrane and actin cytoskeleton of the invadosome.

### βI- and βIII-spectrin chains are specifically enriched in invadosomes

**Fig 2:**
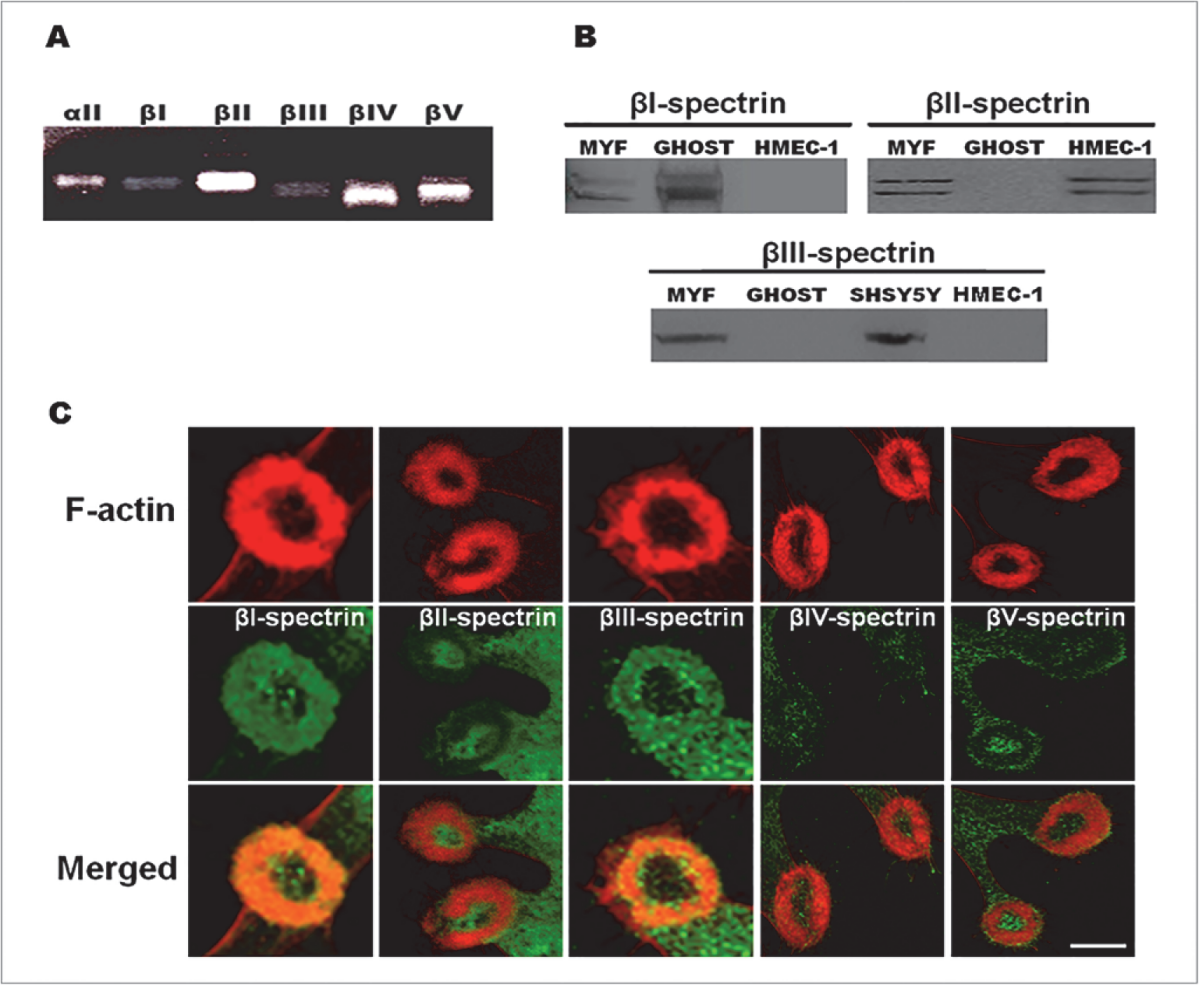
αIIβI- and αIIβIII-spectrin heterodimers are specifically present in invadosomes. (A) RT-PCR products analysis of β-spectrin chains. The presence of β-spectrin mRNAs (βI, βII, βIII, βIV, βV) was investigated using extracted RNA from total lysates of SrcY527F-MEF cells. Higher expression of βII-, βIV- and βV-spectrin chains was found in these cells. (B) Detection of β-spectrin expression at the protein level in SrcY527F-MEF cells (MYF). Western blotting was performed from 20 μg of proteins of MYF cells and compare to a βI-spectrin positive control from a preparation of red blood cells membranes (GHOST, 0,7 μg per lane), to a βII-spectrin positive control from a preparation of HMEC-1 endothelial cells (20 μg per lane) and to a βIII-spectrin positive control from a preparation of SHSY5Y neuronal cells (20 μg per lane). βI-spectrin, conventional βII- and βIII-spectrins were detected in SrcY527F-MEF cells. (C) SrcY527F-MEF cells were fixed and stained for the different β-spectrin chains (green) and invadosome structures were visualized by F-actin staining (red). βI- and βIII-spectrins are specifically enriched in invadosome rings while βII-, βIV- and βV-spectrins are not present. Scale bar: 5 μm.

Spectrins exist in cells mostly as αβ heterotetramers, made of different α and β-spectrins that display distinct tissue-specific cellular and subcellular patterns and functions. As αII-spectrinis specifically enriched in invadosomes, we looked for the β-spectrin isoforms that could be associated with αII-spectrin. RT-PCR experiments with SrcY527F-MEF revealed expression of all β-spectrins mRNAs, with an enrichment of βII-, βIV- and βV-spectrin mRNA (Fig. 2A). As shown by immunoblotting, SrcY527F-MEF cells express βI, βII and βIII spectrins whereas only the βII isoforms could be detected in HMEC-1 cells (Fig. 2B). Membrane preparations of red blood cells (ghost) and a human derived neuroblastoma cell line, the SHSY5Y cells, were used as positive controls for expression of βI-spectrin and βIII-spectrin, respectively. We could not analyze βIV- and βV-spectrins as no reactive bands could be detected with these antibodies in western blots. As SrcY527F-MEF cells express most of the β-spectrin isoforms, they were used to localize each of these β-spectrins in invadosomes (Fig. 2C). βI and βIII-spectrins clearly co-localized with actin and were present in invadosome rings while βII-, βIV- and βV-spectrins were not associated with the invadosomes. These data support that other spectrin chains, βI- and βIII-spectrins are present in the invadosomes while the other β-spectrins are excluded. Thus, invadosome rosettes constitute a microenvironment enriched in specific βI and βIII-spectrins.

### Dynamics of αII-spectrin during invadosome life-span

**Fig 3:**
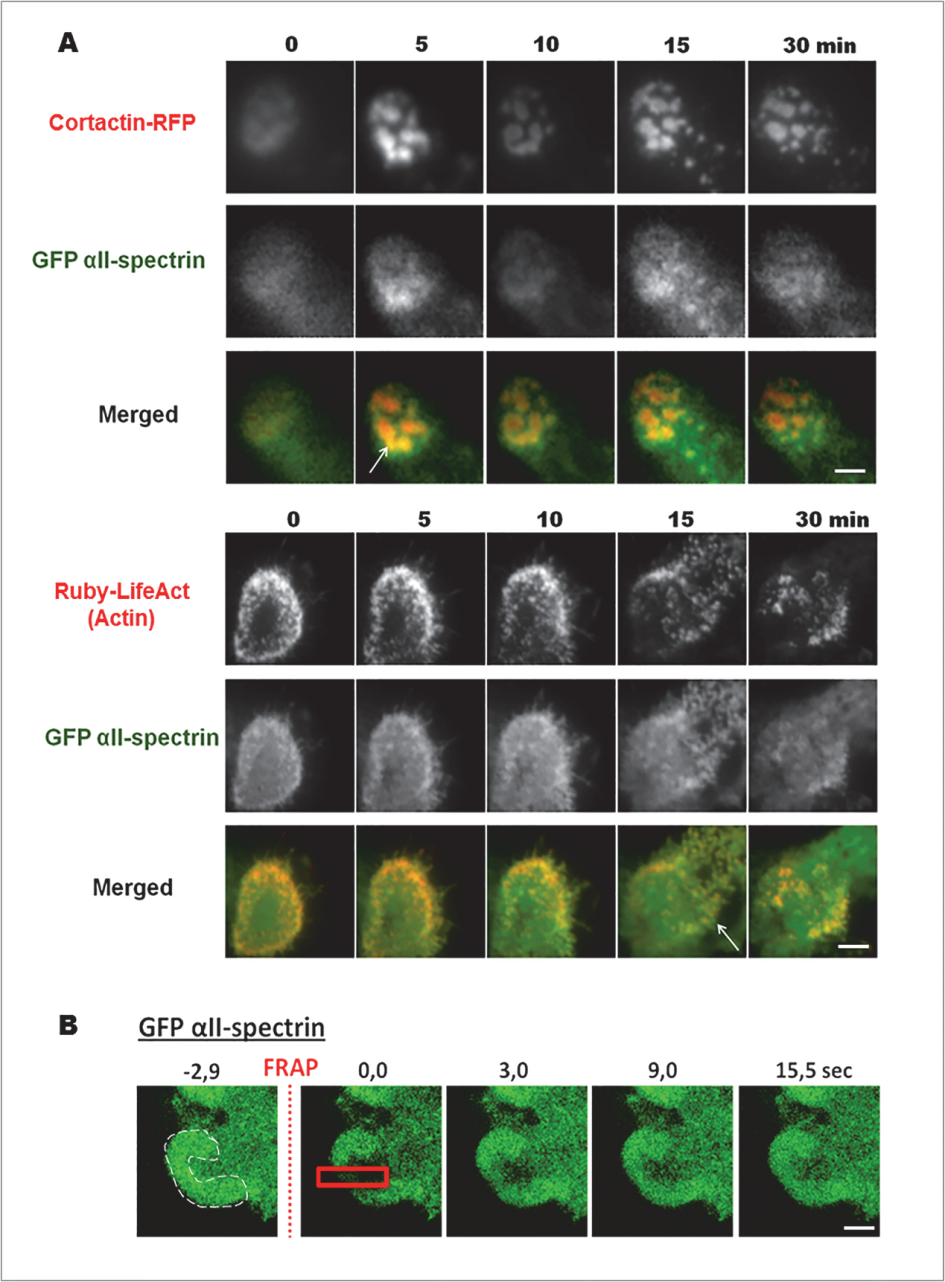
GFP αII-spectrin dynamics in invadosomes. (A) Extracted images from time series (min) from representative observations by TIRF microscopy of living SrcY527F-MEF cells expressing full length of αII-spectrin fused to GFP an invadosome marker fused to RFP, cortactin, or the actin marker Ruby-LifeAct. Ten observation fields were analyzed from at least three different experiments. GFP-αII-spectrin accumulated in isolated or invadosome rings, during their expansion or disorganization. These data link αII-spectrin with the intense actin remodeling associated with invadosome dynamics. (B) To confirm this point, net flux of GFP- αII-spectrin was analyzed by FRAP technology. After a 2.9 sec photobleaching in the red square, αII-spectrin fluorescence starts to reappear after only 3.0 sec, and total recovery of the fluorescence in the photobleached area occurred at 15.0 sec. The invadosome ring is identified by a white dash line here in a portion of cell in the first time. Relevance of the results were obtained by analyzing 10 observation fields from at three different experiments. Scale bar: 3 μm.

To determine the specificity of αII-spectrinrecruitment during a particular phase of invadosome ring dynamics (assembly, steady-state or disassembly), the complete cDNA sequence ofαII-spectrin fused to GFP was co-expressed with invadosome markers: cortactin fused to RFP and the LifeAct peptide fused to Ruby that links polymerized actin in SrcY527F-MEF cells. These living cells were imaged by TIRF microscopy, which gave excellent signal to noise ratio for GFP αII-spectrin and allowed us to specifically observe the basal membrane vicinity. Cells overexpressing αII-spectrin were viable and did not exhibit any shape modifications. As the endogenous protein, GFP αII-spectrin was found in invadosome units as well as in ring metastructures. GFP αII-spectrin accumulation was not observed prior to formation of the new invadosome, showing that this molecule does not seem to be involved in the initial formation of these structures. During invadosome disorganization, αII-spectrin did not accumulate after complete disorganization of the invadosome, but rather followed Ruby-LifeAct or cortactin-RFP behaviors, suggesting that GFP αII-spectrin dynamics correlated more to the actin dynamics than to the invadosome disorganization (Fig. 3A). Moreover, it has been demonstrated that invadosomes present another level of dynamics: while the ring does not move (steady-state), the F-actin core is renewed multiple times within the invadosome unit [23]. To check if the internal dynamics of αII-spectrin correlated to the continuous renewal of actin polymerization occurring during the steady-state phase of the invadosome cycle than to the slow mobility of adhesion receptors such as integrins, we used the FRAP technology to determine the molecular mobility of GFP αII-spectrin in invadosomes (Fig. 3B). After photobleaching of a small region in an invadosome ring (Fig. 3B, red square), the fluorescence recovery of GFP αII-spectrin occurred within few seconds after illumination and have a characteristic time of recovery around 10 sec. Based on this constant exchange between spectrin molecules in the invadosome, our results showed that the immobile fraction of GFP αII-spectrin is minimal, and that this protein has an important turnover rate close to that observed for actin-GFP [23]. According to its localization and its dynamics, αII-spectrin may function in conjunction with the specific organization of actin in the invadosomes.

### Knockdown of αII-spectrin impairs invadosome formation and architecture

**Fig 4:**
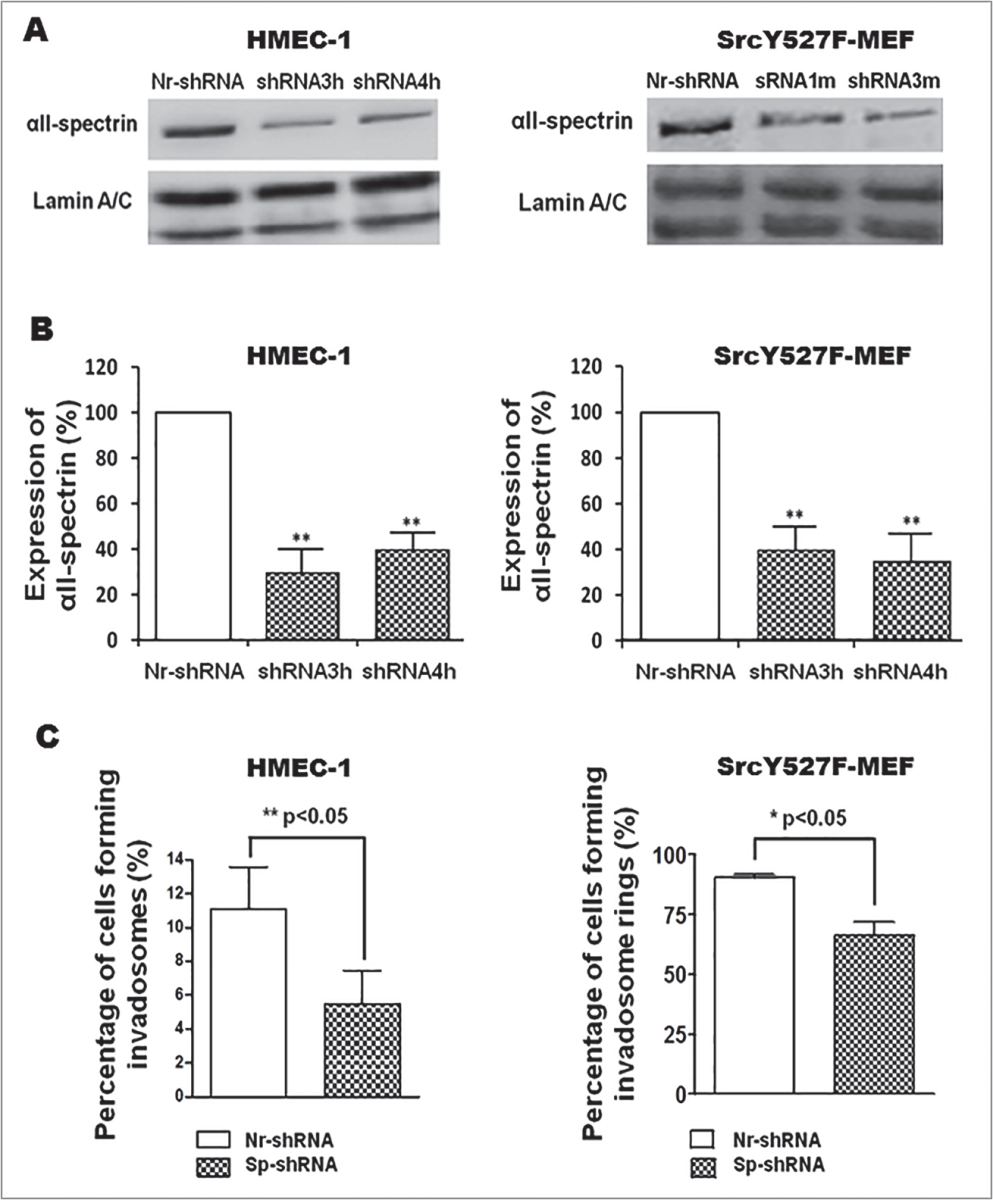
αII-Spectrin silencing revealed its role in invadosome formation. SrcY527F-MEF and HMEC-1 cells were transfected for 96 hr with different shRNA directed against αII-spectrin (Sp-shRNA) or an irrelevant shRNA (Nr-shRNA). (A and B) Western Blot analysis revealed a 60% decrease in αII-spectrin expression. Lamin A/C was used to control protein loading. Results revealed efficiency of shRNA3h and 4h (for HMEC-1), 1m and 3m (for SrcY527F-MEF) on αII-spectrin expression. (C) Invadosomes were counted 96 hr after cell transfection with shRNA (GFP positive cells): 300 transfected cells were counted from at least 3 independent experiments. The percentage of cells forming invadosomes was evaluated in control cells (Nr-shRNA) versus spectrin-depleted cells (Sp-shRNA). αII-Spectrin silencing significantly reduced the percentage of HMEC-1 and SrcY527F-MEF cells forming invadosomes.

**Fig 5:**
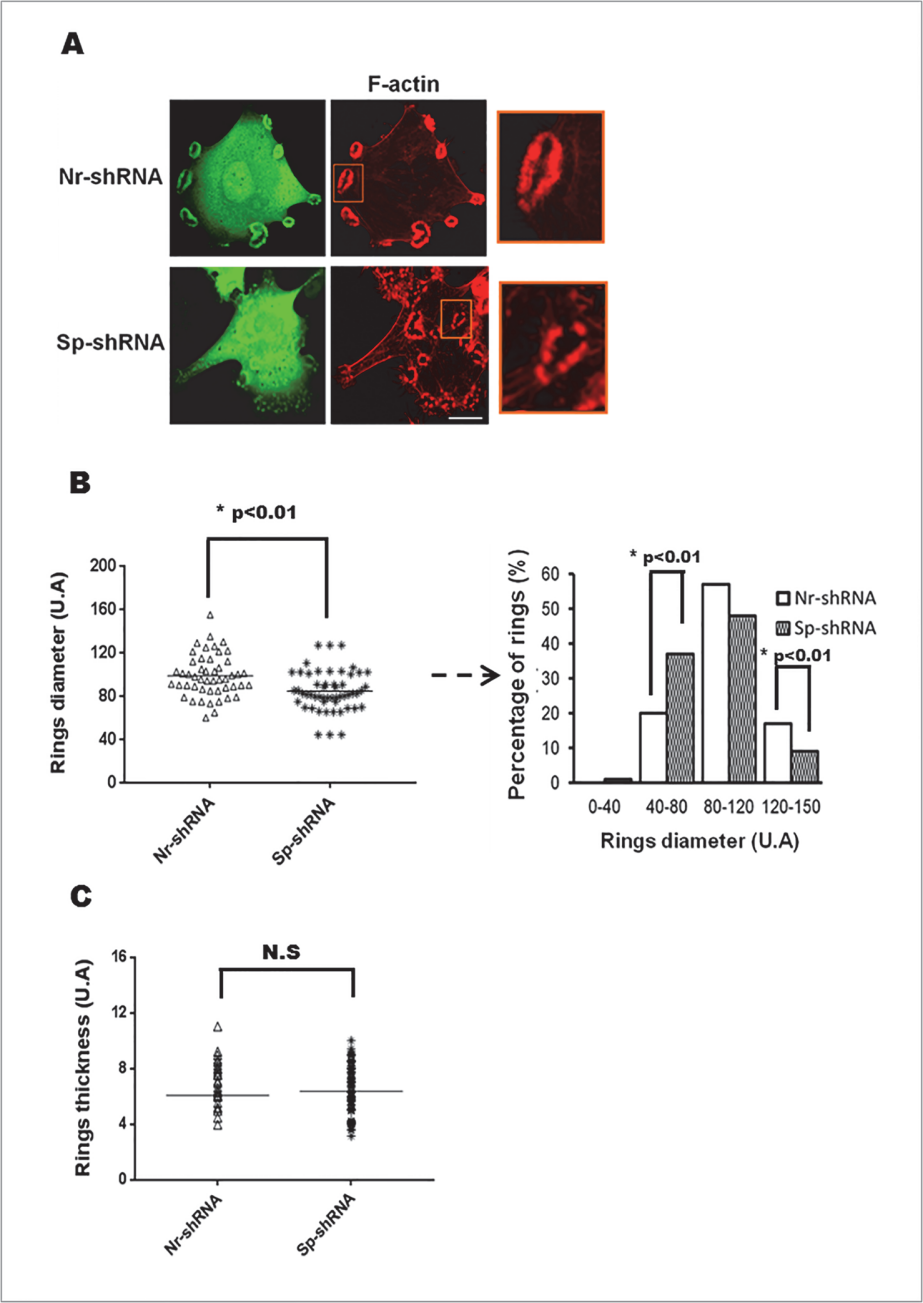
αII-Spectrin silencing changes invadosome morphologies. SrcY527-MEF cells were transfected for 72 hr with shRNA αII-spectrin (Sp-shRNA) or irrelevant shRNA (Nr-shRNA). (A) F-actin of transfected cells was stained with phalloidin toxin (red) allowing to visualize invadosome rings. Analysis of cells (10 fields per condition) revealed an increased number of fragmented invadosome rings in spectrin-depleted cells. (B) Invadosome ring diameters and (C) invadosome ring thickness were measured in transfected cells. Around 100 rosettes (corresponding to 20 cells, chosen in random field) were analyzed per conditions in 3 different experiments. Knockdown of αII-spectrin induces a significant decrease in ring diameters due in particular to an increase in the percentage of rings with a small size and a decrease number of large rings. In another side αII-spectrin depletion does not modify rings thickness. Scale bar: 20 μm.

After investigating the dynamic presence of αII-spectrinin invadosomes, its function(s) was further explored by silencing its expression in HMEC-1 and SrcY527-MEF cells using short hairpin RNA (shRNA) technology. Cells were transfected with control shRNA (Nr-shRNA) or different shRNAs targeting either mouse (m) or human (h) αII-spectrin mRNAs, i.e., shRNA1m or 3m for SrcY527F-MEF and shRNA 3h and 4h for HMEC-1. As shown by western blot analysis, 96 h after transfection, these different shRNAs efficiently silencedαII-spectrin expression by 60% (Fig. 4A and B). As shRNA plasmids expressed GFP, the impact of αII-spectrin depletion on invadosome formation and morphology was evaluated in GFP positive cells. Quantitative analysis showed that depletion of αII-spectrin significantly reduced the number of cells forming invadosomes (isolated and rings) by 60% in HMEC-1 cells and by 30% in SrcY527F-MEF compared to cells treated with an irrelevant shRNA (Fig. 4C). This result supports the involvement of αII-spectrin in invadosome formation or stabilization. Because they form numerous invadosomes, SrcY527F-MEFs have been used to facilitate morphometric analysis on the remaining invadosome structures. Even though silencing αII-spectrin expression decreased the percentage of cells forming invadosomes, the αII-spectrin-depleted cells exhibited a 45% increase in the number of ring structures (Nr-shRNA 11.85 ± 0.5471 vs. Sp-shRNA 16.82 ± 0.9819 p<0.01). The invadosome rings remaining after αII-spectrin silencing appeared smaller and fragmented, as evidenced by the decrease of invadosome ring diameter after morphometric analysis and the shift in distribution of ring diameters (Fig. 5A and B), whereas the mean thickness of the rings was not altered (Fig. 5C). These morphological changes were not associated with changes in the expression and localization of the αII-spectrin partners implicated in actin regulation such as ABI1, VASP, and WASL (S2 Fig.). Moreover, αII-spectrin knockdown did not change the localization, expression or membrane recruitment of the two main key signalling regulators of invadosomes, Src and PKC (S3 Fig.). Overall, the results suggest that αII-spectrin may have a role in invadosome dynamics, function and ring expansion.

### αII-Spectrin knockdown disturbs invadosome-mediated matrix degradation and cellular invasion

**Fig 6:**
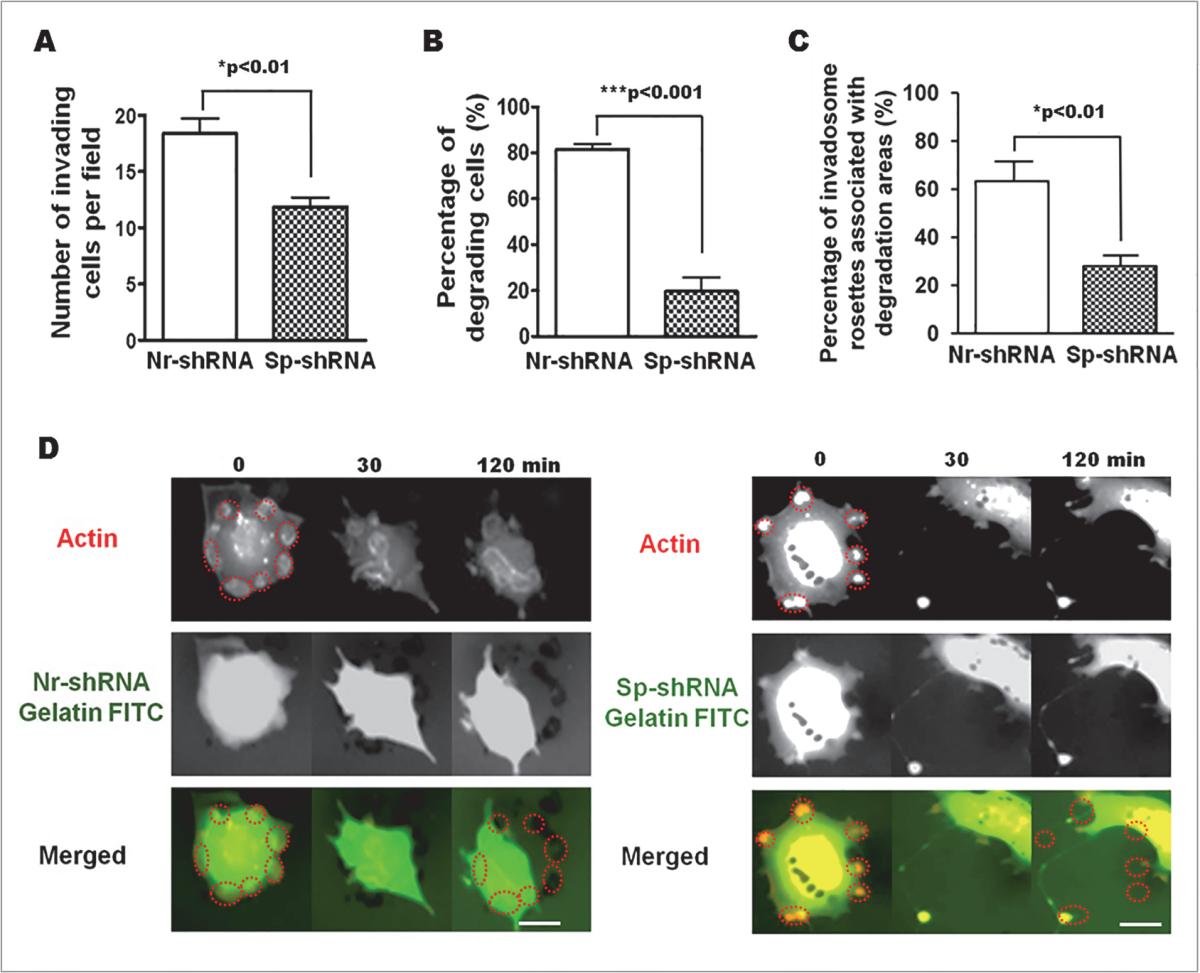
Knockdown of αII-spectrin decreases cell invasion and matrix degradation activity of invadosomes. (A) SrcY527F-MEF were transfected for 72 hr with shRNAs (Nr-shRNA and Sp-shRNA) and seeded on Boyden’s chamber coated with a layer of Matrigel in order to obtain an invasion chamber. Around 15 random fields were analyzed per condition and results are presented as pool of three different experiments. After 20 hr, the number of invasive cells was significantly reduced by 35% when spectrin was silenced (Nr-shRNA 18,40 vs. Nr-shRNA 11,85 p<0,01). (B) This decrease of the invasive properties of SrcY527F-MEF depleted for αII-spectrin was associated with a large decrease in the percentage of cells able to digest an extracellular matrix component such as FITC-gelatin (Nr-shRNA 81.53 ± 2.31% vs. Sp-shRNA 19.67 ± 6.17%; p<0,001). (C) More precisely, the percentage of invadosome rings associated with a degradation area was significantly reduced in spectrin-depleted cells (Nr-shRNA 63.25 ± 6.70% vs. Sp-shRNA 28.15 ± 3.00%; p<0.01). (D) Extracted images from time series (min) from representative observations of living SrcY527F-MEFcells transfected for 72 hr with shRNAs (Nr-shRNA and Sp-shRNA) and Ruby-LifeAct. Spectrin depletion impairs matrix degradation as shown by the lack of black areas (Sp-shRNA, 120 min) in the red circles, indicating invadosomes at time 0. Scale bar: 20 μm.

As invadosomes are involved in ECM degradation and cell invasion, these functional properties were investigated inαII-spectrin-depleted cells. Cell invasiveness was determined by measuring the number of cells able to pass through a thin layer of gelatin coating in a Boyden chamber. Control (Nr-shRNA) and spectrin-depleted (Sp-shRNA) SrcY527F-MEF cells were seeded on these chambers. After 24 hr, spectrin-depleted cells displayed a significant defect in invasiveness as manifested by a 35% decrease in the number of cells able to invade the gelatin (Fig. 6A).

In this assay, the invasion process involves both cell migration and ECM layer digestion. To explain the decreased invasion ability of αII-spectrin-depleted SrcY527F-MEF cells, matrix degradation was determined using 2D gelatin-FITC surfaces. Static analysis revealed that only 20% of αII-spectrin-depleted cells were able to degrade the matrix. The number of invadosomes associated with a digested area was 60% lower in spectrin-depleted cells than in control cells (Fig. 6B and C). These data show that αII-spectrin could play a role in coupling actin reorganization and ECM degradation. To answer this question, the degradation activity of invadosomes was determined on liveSrcY527F-MEF cells, spread on a digestible gelatin-FITC layer. The SrcY527F-MEFs are co-expressing the actin invadosome marker, Ruby-LifeAct, and GFP, which is the reporter of the Nr-ShRNA or Sp-ShRNA vectors. Although GFP is masking the gelatin-FITC signal, invadosome positions could still be marked and recorded over time (Red dashed circles, Fig. 6D). The migration of the cells out of these areas allows observing the gelatin layer corresponding to the previously marked and recorded invadosome positions. Under control conditions (Nr-shRNA), black spots were detected at the end of the visualization (Fig. 6C, 120 min), corresponding to invadosome localization at time 0 (Fig. 6D, red dashed circles) whereas no black spots co-localized with invadosomes of spectrin-depleted cells. This matrix degradation defect phenotype is not associated with a change of MMP2 and MMP9 secretion, as their expression measured by western blot and their secretion assessed by zymography were not significantly modified (S4 Fig.). Additionnally, the transmembrane MT1-MMP (MMP14) metalloprotease present a slight decrease expression in αII-spectrin depleted cells (S4 Fig.). Altogether, both static and dynamic analyses showed that αII-spectrin depletion allows the formation of very dynamic invadosomes, but alters ECM degradation. Thus, this clearly demonstrates the involvement of αII-spectrin in the coupling between matrix degradation and actin reorganization in invadosomes.

### αII-Spectrin regulates invadosome dynamics and stabilizes β3-integrin anchorage

**Fig 7:**
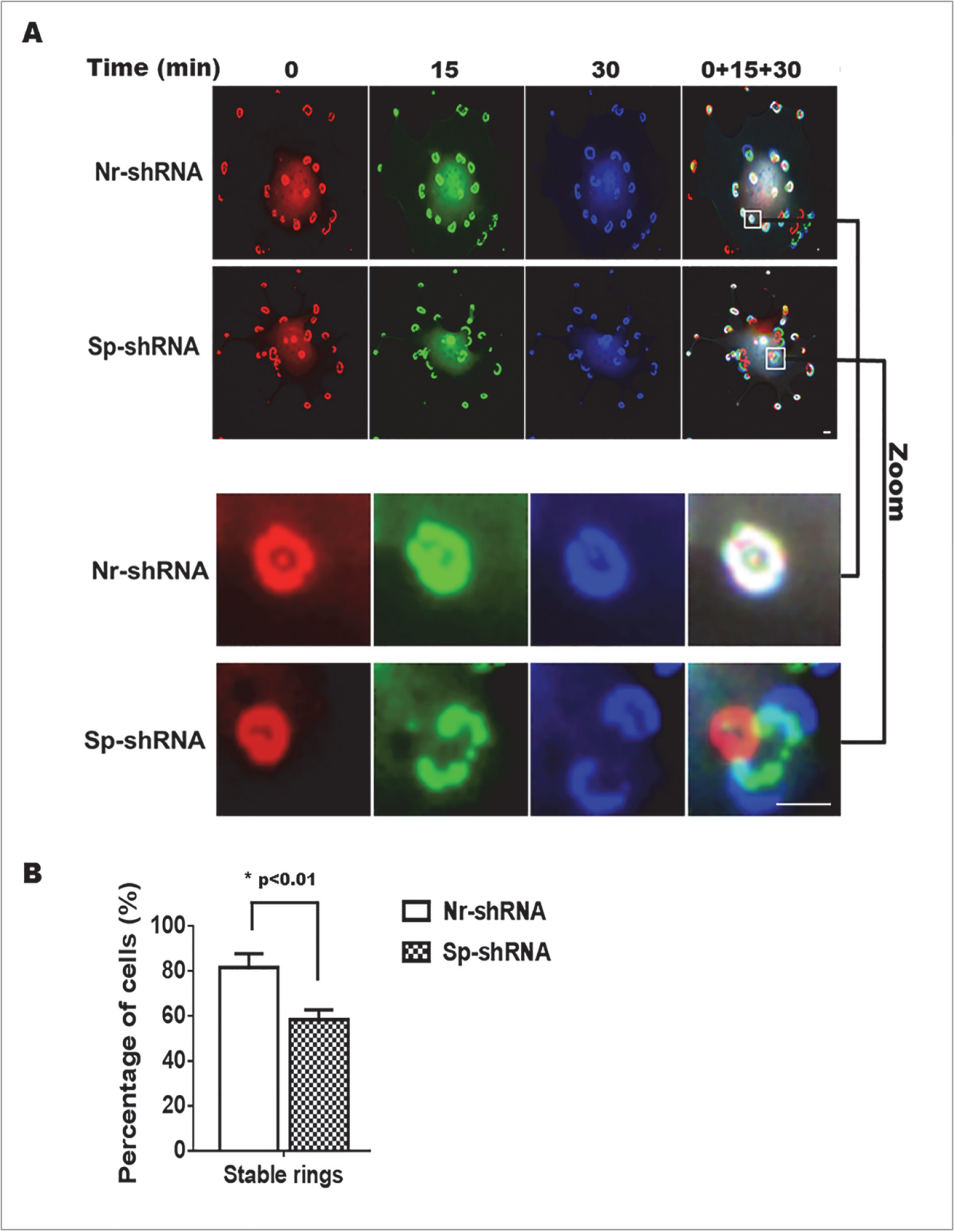
Knockdown of αII-spectrin stimulates the formation of hyperdynamic invadosome rings. SrcY527F-MEF cells were co-transfected with plasmid encoding an irrelevant shRNA (Nr-shRNA) or a shRNA targeting αII-spectrin (Sp-shRNA) and Ruby-LifeAct to follow invadosome dynamics. (A) Spectrin-depleted cells present hyperdynamic invadosome rings as showed by video microscopy analysis. Images taken at three different times (0, 15 and 30 min) and pseudo colored in red, green and blue revealed stable invadosome by white colored structures while dynamic invadosomes are not at the same position in these three different times. (B) In the control cells, a majority of cells presents stable invadosome rings (85%) over the 30 min observation, while only 15% of them change (dynamic movement and disorganization) over this period. On the contrary, spectrin depletion increases the percentage of cells with hyperdynamic invadosome rings over this period of observation (40%) and consequently decreases the global stability of invadosome rings (60%). The dynamics of invadosomes was analyzed in 30 to 40 cells per condition in three different experiments. Scale bar: 5 μm.

To understand how the greater number of invadosome rings can be associated with a decrease ability to degrade the ECM, we investigated the impact of αII-spectrin knockdown on invadosome dynamics. The behavior of invadosomes in live SrcY527F-MEF cells co-transfected with Ruby-LifeAct and either Nr-shRNA (control) or shRNA targeting αII-spectrin was visualized by videomicroscopy. Based on 30 min recordings, invadosomes in all cells exhibited two types of behavior, i.e., stable invadosome rings or sliding invadosomes (Fig. 7A). To better understand these behaviors, invadosome images taken at different times (0, 15 and 30 min) were colorized with assigned colors and merged. Stable invadosomes were indicated by white color while unstable rings appeared as concentric multicolor structures. Analysis of records showed that around 85% of control cells (Nr-shRNA) exhibited a majority of stable invadosome rings over 30 min (Fig. 7B). In contrast, only 60% of spectrin-depleted cells showed stable rings (Nr-shRNA 82±5% vs. Sp-shRNA 59±3% p<0.01). Thus, depletion of αII-spectrin increases the formation of unstable invadosome rings that are poorly able to digest the ECM. These results show that a decrease in αII-spectrin expression leads to uncoupling between invadosome dynamics and ECM degradation.

**Fig 8:**
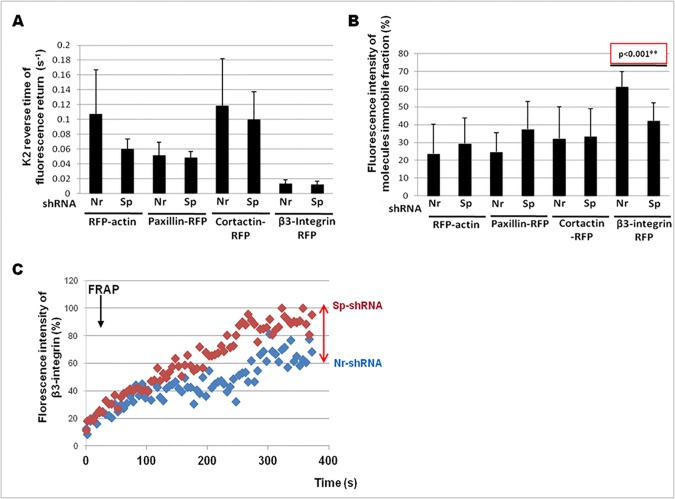
Deficiency of αII-spectrin increases the mobile fraction of β3-integrin in invadosome rings. SrcY527F_MEF cells were transfected for 72 hr with irrelevant shRNA (Nr-shRNA) or shRNA targeting αII-spectrin (Sp-shRNA) along with RFP-actin, paxillin-RFP, RFP-cortactin or β3-integrin RFP plasmids in order to quantify the mobility of these invadosome components (by FRAP) in presence or absence of αII-spectrin. (A) A simple exponential equation model was used to quantify K2 (in sec^-1^), which is the inverse of the characteristic time of fluorescence recovery (tau), in order to evaluate the diffusion speed of these invadosome proteins. No significant changes were observed, indicating that αII-spectrin does not affect the net rate of entry of these components in invadosomes. (B) Quantification of the immobile fraction of each of these invadosome components. Immobile fraction of actin, paxillin and cortactin remained constant between control and spectrin-depleted cells. However, αII-spectrin depletion significantly reduced the immobile fraction of β3-integrin in invadosomes. (C) Representative FRAP recovery curves of β3-integrin-RFP in presence (Nr-shRNA) or absence (Sp-shRNA) of αII-spectrin. In absence of αII-spectrin, the number of immobile β3-integrin molecules is more important while the plateau is smaller than in presence of αII-spectrin. Results for quantification were obtained from 3 independent experiments where 10 to 11 cells were analyzed for each experiment.

In order to understand the instability of invadosome rings in αII-spectrin depleted cells, we hypothesized that αII-spectrin could affect the dynamics of invadosome regulators at the plasma membrane. Thus, we analyzed the dynamics of four invadosome components, two implicated in actin dynamics (actin, cortactin) and two in invadosome adhesion (paxillin, β3-integrin. For that purpose, we used the FRAP technology to determine potential perturbation in the mobility of these proteins in absence of αII-spectrin (Fig. 8). Through the use of the FRAP module of Zen blue software (Carl zeiss imaging), protein mobility was analyzed by measuring the average characteristic time of fluorescence recovery (Fig. 8A), and the average immobile fraction corresponding to the fraction of molecules that cannot exchange between bleached and non-bleached regions (Fig. 8B) for each molecule present in an invadosome ring. First, αII-spectrin depletion did not induce any change in the rate of net exchange (characteristic time of recovery) of any molecules tested. Surprisingly, the net flux of actin-RFP was not modified in the remaining invadosome rings induced by αII-spectrin depletion (Fig. 8A). However, analysis of the immobile fraction showed that αII-spectrin silencing in SrcY527F-MEF cells induces a 60% decrease in the immobile fraction of the β3-integrin in invadosome rings (Fig. 8B). Thus, αII-spectrin regulates the amount of β3-integrins in invadosomes, but does not alter the β3 exchange rate (Fig. 8C). As a control, we noticed that the depletion of αII-spectrin did not modify the immobile fractions of actin, paxillin, or cortactin. Moreover, no modifications of total expression or cell surface expression of β1- and β3-integrins were observed in αII-spectrin-depleted cells (S5 Fig.). FACS analysis revealed also that β1-integrin activation was not changed (S5 Fig.).

Thus, besides being dynamically associated with actin, αII-spectrin does not modulate directly the actin cytoskeleton, but rather seems to be a link to adhesion receptors while regulating the immobilization of β3-integrins in invadosomes.

## Discussion

Thus far, more than thirty proteins have been identified in invadosomes. Our study investigated the function of a poorly known component of invadosomes, αII-spectrin. Most of spectrin functions have been determined from studies in red cells, and have clearly established its importance in supporting cell shape and plasma membrane stability [2, 3]. In non-erythroid cells, the functions of spectrins are less clear, especially in the regulation of ECM adhesion structures. In this study, we demonstrate the presence of αII-spectrin in invadosomes from HMEC-1 and HUVEC endothelial cells stimulated by phorbol ester (PMA) and in MEF cells transformed by SrcY527F. Moreover, invadosomes were enriched in αIIβI- and αIIβIII- spectrins. Live cell imaging revealed the intense dynamics of αII-spectrin in invadosomes while silencing experiments revealed its role in the regulation of the formation of invadosome rings and their degradation activity. Silencing of αII-spectrin was also associated with a defect in cell migration leading to severe inhibition of cell invasion and a decrease in stabilization of β3-integrins in the invadosomes, supporting a role for αII-spectrin in these cellular functions.

### αII-Spectrin is a component of invadosomes

The presence of αII-spectrin was reported in podosome-like structure of non-invasive squamous carcinoma cells, but not in invadopodia of invasive cells [18]. Our experiments show that αII-spectrin silencing is associated with a decrease in ECM-degradative activity and consequently with a decrease of cellular invasion. Spectrin exists as αβ heterodimers, displaying distinct tissue-specific cellular and subcellular patterns of expression. In red blood cells, spectrin is exclusively present as αI/βI subunits, as compared with nucleated cells where the αII chain is associated with βI to βV chains, while αII and βII chains are mostly expressed in all non-erythroid cells, and the other spectrin isoforms have more specific distributions. The present study reports for the first time the presence of βI and βIII-spectrins in invadosomes while βII, βIV- and βV-spectrins were excluded from the invadosomes rings, suggesting a specific role for each isoform. Whereas αII-spectrin could not be found in focal adhesions, we show its presence in invadosomes. Even though invadosomes are close to focal adhesions in terms of protein composition [25], they are structurally distinct by their dynamics and their specific 3D actin organization, represented by an inner actin core, perpendicular to the substratum, and surrounded by an actin cloud [40]. Spectrins have been described in some actin-rich and adhesive structures involved in cell-cell contacts. The common point between invadosomes and cell-cell contact structures is their dependency on intense actin remodeling. This suggests that αII-spectrin function in adhesion (cell-cell and cell-ECM) should be rather seen as a link between actin architecture and adhesion receptors.

### αII-Spectrin modulates formation, architecture and stability of invadosome structures

The percentage of cells forming invadosomes is reduced by the knockdown of αII-spectrin. However, the remaining invadosomes are mostly organized in rings, which exhibit structural modifications of their shape (fragmented structures and modifications of both size and thickness). These data suggest that αII-spectrin may stabilize the invadosomes. We consider the functional link between αII-spectrin and F-actin. Indeed, αII-spectrin is an actin-binding protein, which interacts via its SH3 domain with proteins such as ABI1 [41], VASP [42] and N-WASP [43] that actively participate in the regulation of actin polymerization in invadosomes. αII-Spectrin co-localized with ABI1, VASP and N-WASP in invadosomes, but no change of localization or expression of these proteins were observed in SrcY527F-MEF cells depleted of spectrin. However, αII-spectrin silencing induced perturbations in the invadosome architecture while no modification of the net flux of polymerized actin was observed (Fig. 7). Thus, αII-spectrin does not seem to be involved in actin regulation in invadosomes. As invadosome rings in αII-spectrin-depleted cells exhibit an abnormal dynamic behavior, we believe that αII-spectrin might act on actin remodeling by rather playing a role in the coupling between F-actin and the integrin adhesion proteins highly present in invadosomes. One main characteristic of the invadosomes in SrcY527F-MEF spectrin-depleted cells is their instability (fusion/fission) and very dynamic behavior. This led us to consider that αII-spectrin rather alters the adhesive property of integrins in invadosomes.

### Spectrin functions in integrin mobility

Integrins are recruited in invadosomes and play an important role in the regulation of invadosome functions [44–47]. In SrcY527F-MEFs, β1- and β3-integrins are associated with invadosomes. While β1-integrin is essential for the formation and organization of invadosomes, β3-integrin seems to have a less essential function in the initiation of invadosome formation [35]. The link between αII-spectrin and integrins could be based on general function of spectrin in addressing and/or stabilization of many membranous proteins at the plasma membrane, including adhesion molecules such as Lu-BCAM and NCAM-180. Spectrin plays a role in the adhesion of Lu-BCAM to α5-laminin [48–50], and is involved in the stabilization of NCAM180 at the membrane [51]. Members of the protein 4.1 family may also have a role as indirect mediators of spectrin-integrin interaction. Protein 4.1 is a spectrin- and actin-binding protein that controls the cell surface accumulation and function of certain beta-integrins [52].

Previous work demonstrated that a human melanoma cell line depleted in αII-spectrin induced adhesion defects associated with modified α5- and αVβ3-integrins localization [8]. Moreover, knockdown of αII-spectrin affected the adhesion of SrcY527F-MEF (S5A Fig.) and HMEC cells (Ponceau and Lecomte, personal communication), inducing a slower adhesion kinetic as compared to control cells (S5A Fig.). Our data suggest that the instability of the invadosomes in αII-spectrin-depleted cells could be due to a perturbation of adhesion. Until now, there are no biochemical data suggesting a direct link between spectrin and integrins, but some results support an indirect link. Indeed, data show that the SH3 domain of αII-spectrin is recruited in β3-integrin clusters [53] and can delay adhesion on vitronectin. This link between integrins and αII-spectrin is further supported by the fact that depletion of αII-spectrin decreased the number of β3-integrins immobilized in invadosomes (Fig. 8). Thus, it appears that αII-spectrin could be a physical link between adhesion complexes and F-actin polymerization occurring in invadosome. The recruitment of αII-spectrin could then facilitates the recruitment of β3-integrins in an intense F-actin polymerization region as invadosome and then increases the transmission of the forces generated by this polymerization to the extracellular matrix.

### Spectrin is involved in matrix degradation and consequently in cell invasion

These defects in the adhesion process could also explain the defect of ECM degradation. Defect in the cycle of activation of β1-integrin is known to inhibit ECM degradation by SrcY527F-MEFs [35]. Moreover, β3-integrin ^(-/-)^ SrcY527F-MEF cells are also defective in ECM degradation (Destaing, personnal communication). The link between integrins and αII-spectrin is again supported by the fact that knockdown of αII-spectrin in SrcY527F-MEFs decrease ECM degradation activity of invadosomes. Decrease of β3 immobilization induced by αII-spectrin depletion is associated with a defect of adhesion on vitronectin (S5A Fig.). This decrease of β3-dependent adhesion due to less immobile integrins is correlated with an increase of invadosome rings dynamic in αII-spectrin depleted cells. Thus, the decrease of ECM proteolysis observed in αII-spectrin depleted cells could be explained by a decrease of a β3-dependent anchoring of invadosomes that will be then less efficient to digest the ECM. In addition to this defect in ECM degradation, αII-spectrin depletion leads also to a decrease of cell migration, indicating that targeting αII-spectrin could be essential to control cell invasion.

In summary this study reports a novel function ofαII-spectrin, the major protein of membrane cytoskeleton, as a component of invadosome structures. The data show that αII-spectrin plays a critical role in invadosome stability and β3-integrin stabilization. In this manner, αII-spectrin participates in important invadosome functions such as cell adhesion and migration as well as ECM degradation and invasion.

## Supporting Information

S1 Fig αII-Spectrin is found in invadosomes induced by growth factor stimulation. Starved HMEC-1 cells were treated for 1 hr with EGF (5 ng/ml, A) or TGFβ (5ng/ml, B) in order to induce characteristic invadosome rings (zoom red square). Then, endogenous cortactin and αII-spectrin were stained and αII-spectrin relocalization was visualized after invadosome induction. Scale bar: 10 μm.
(TIF)

S2 Fig Recruitment and expression of actin-regulatory proteins are not affected in αII-spectrin-depleted SrcY527F-MEF cells. (A) SrcY527F6 MEF cells were transfected for 96hr with Nr-shRNA or Sp-shRNAs (1m or 3m) and stained for ABI1, VASP and WASL proteins (red). White squares enlarge invadosomes of non-transfected cells, while blue squares enlarge invadosomes of spectrin-depleted cells. Knockdown of αII-spectrin does not affect global distribution of ABI-1, VASP and WASL. (B) Expression of ABI1, VASP and WASL were similar in both control (Nr-shRNA) and spectrin-depleted cells (Sp-shRNA). Lamin A/C was used to control for protein loading. Scale bar: 20 μm.
(TIF)

S3 Fig αII-Spectrin depletion does not affect localization, expression and membrane recruitment of invadosome components. (A) SrcY527F-MEF cells were transfected for 72 hr with shRNA plasmids (Nr-shRNA or Sp-shRNA 1m or 3m) and revealed by the GFP-expression associated with shRNA expression (green). These cells were stained for cortactin, paxillin, phospho-Fak, phospho-cortactin, and protein kinases, Src and PKC (red). After αII-spectrin depletion, no significant changes were observed. (B) PKC and Src expression was not changed and neither was the localization in membranes (Mb), cytosolic (Cy), nuclear (Nu) and cytoskeletal (Ck) fractions. Scale bar: 20μm.
(TIF)

S4 Fig Decrease of matrix degradation activity is not related with metalloproteinases defects. (A) Western blot showing expression of MMP2, 9 and 14. 72 h after transfection with shRNA plasmids (Nr-shRNA, shRNA 1m, shRNA 3m): 20 μg of protein from total lysates of cells were analyzed. (B), Representative zymogram of secreted MMP2 and MMP9. Control and depleted cells were serum-starved during 24 h, then secreted MMPs were quantified in culture supernatants by zymography. Spectrin depletion does not induce significant effects on metalloproteinases secretions.
(TIF)

S5 Fig Spectrin deficiency induces adhesion delay without modifying expression and clustering of invadosomal integrins. (A) SrcY527F-MEF cells were transfected for 96 h with shRNAs (Nr-shRNA, shRNA1m and 3m) and then seeded (100.000 cells) on plastic or vitronectin coated surface. At 10, 20, 30, 60 and 120 min, cells were gently washed and fixed, and the remaining cells corresponding to adherent cells were evaluated. (B) SrcY527F-MEF cells were transfected for 72 hr with irrelevant shRNA (Nr-shRNA) or αII-spectrin shRNAs (shRNA1m and 3m), and cell surface expression of β1-integrin, β3-integrin and an activated form of β1-integrin was analyzed by flow cytometry. αII-Spectrin silencing does not change significantly cell surface expression and activity of these integrins. (C) SrcY527F-MEF cells were transfected for 72 hr with irrelevant shRNA (Nr-shRNA) or αII-spectrin shRNAs (shRNA1m and 3m), and total expression of β1-integrin and β3-integrin was determined by western immunoblotting. αII-Spectrin silencing does not change significantly the expression of these integrins.
(TIF)
