# Supplementary figures and images for: αII-Spectrin Regulates Invadosome Stability and Extracellular Matrix Degradation
Source: PLoS One. 2015 Apr 1;10(4):e0120781. doi: 10.1371/journal.pone.0120781 (PMC4382279; doi:10.1371/journal.pone.0120781)

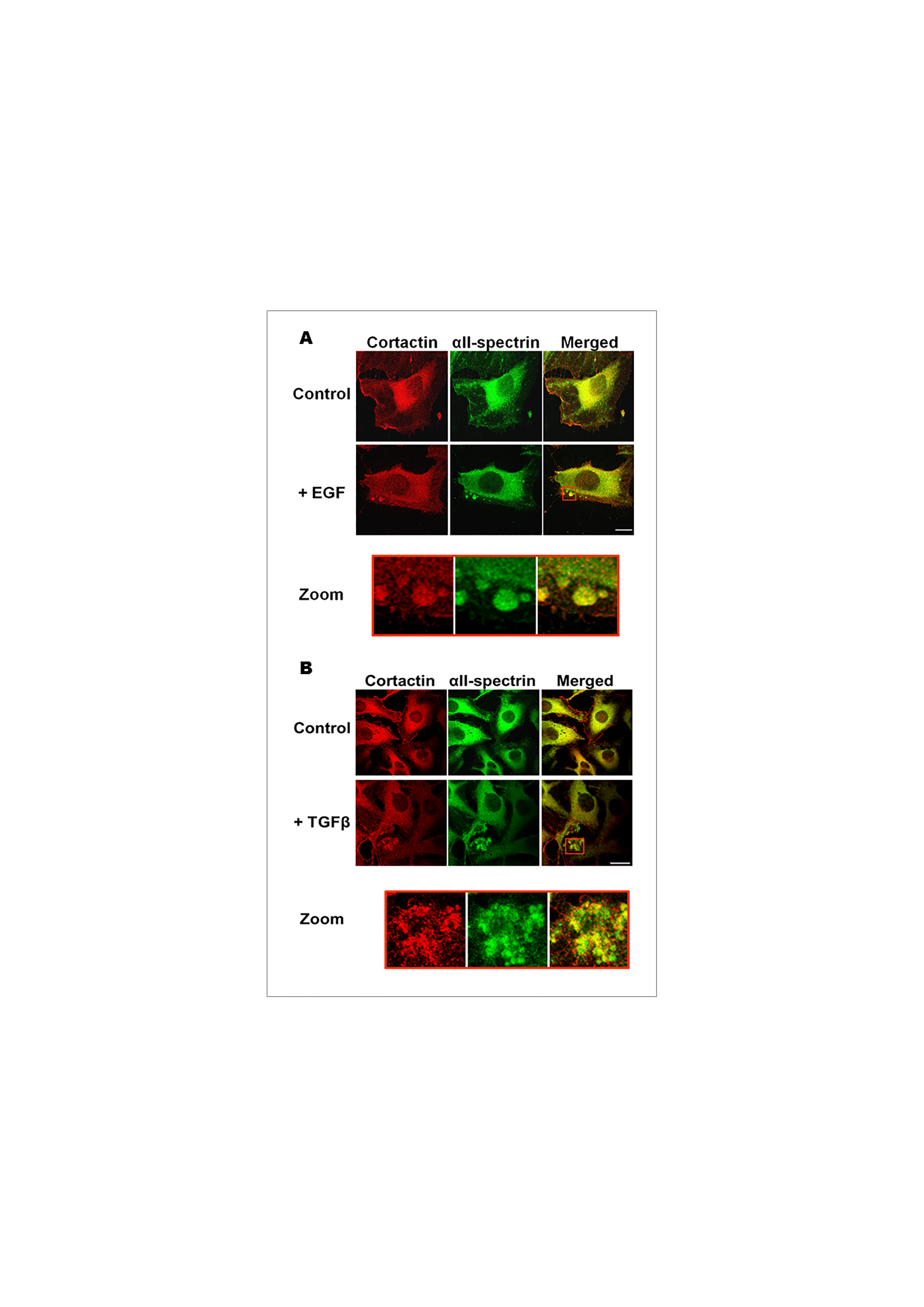

Supplement: S1 Fig — Starved HMEC-1 cells were treated for 1 hr with EGF (5 ng/ml, A) or TGFβ (5ng/ml, B) in order to induce characteristic invadosome rings (zoom red square). Then, endogenous cortactin and αII-spectrin were stained and αII-spectrin relocalization was visualized after invadosome induction. Scale bar: 10 μm. (TIF) [file pone.0120781.s001.tif]

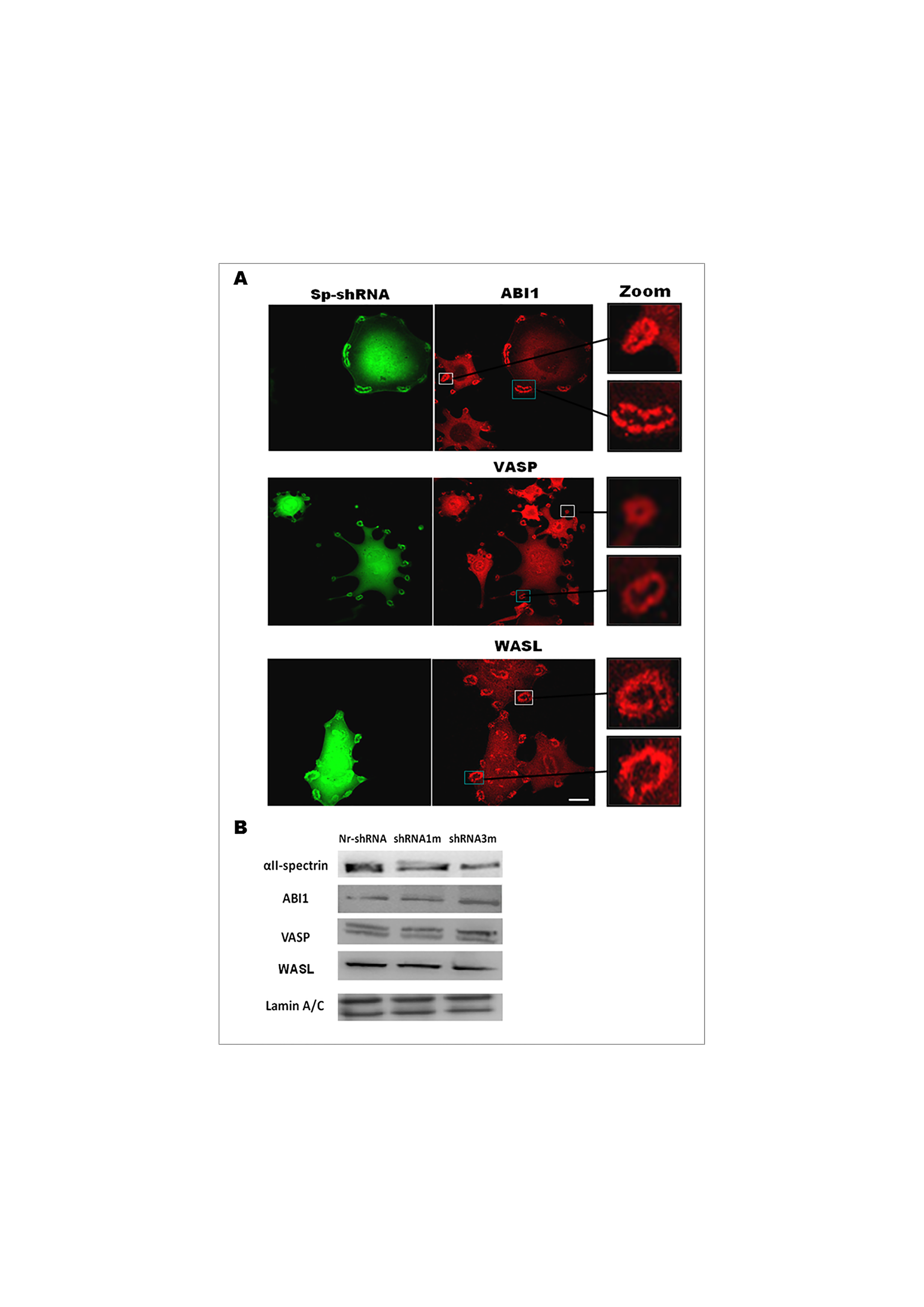

Supplement: S2 Fig — (A) SrcY527F6 MEF cells were transfected for 96hr with Nr-shRNA or Sp-shRNAs (1m or 3m) and stained for ABI1, VASP and WASL proteins (red). White squares enlarge invadosomes of non-transfected cells, while blue squares enlarge invadosomes of spectrin-depleted cells. Knockdown of αII-spectrin does not affect global distribution of ABI-1, VASP and WASL. (B) Expression of ABI1, VASP and WASL were similar in both control (Nr-shRNA) and spectrin-depleted cells (Sp-shRNA). Lamin A/C was used to control for protein loading. Scale bar: 20 μm. (TIF) [file pone.0120781.s002.tif]

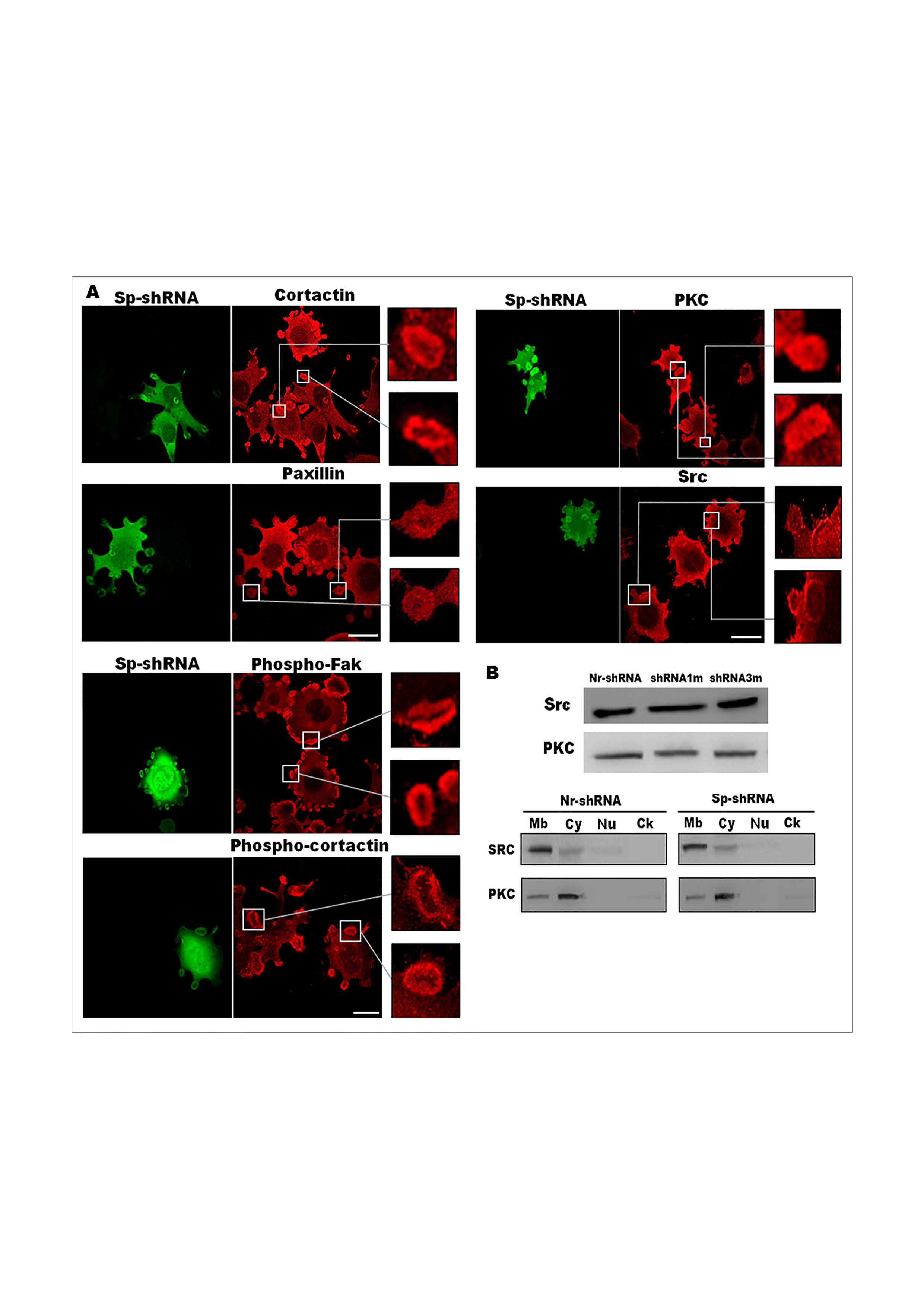

Supplement: S3 Fig — (A) SrcY527F-MEF cells were transfected for 72 hr with shRNA plasmids (Nr-shRNA or Sp-shRNA 1m or 3m) and revealed by the GFP-expression associated with shRNA expression (green). These cells were stained for cortactin, paxillin, phospho-Fak, phospho-cortactin, and protein kinases, Src and PKC (red). After αII-spectrin depletion, no significant changes were observed. (B) PKC and Src expression was not changed and neither was the localization in membranes (Mb), cytosolic (Cy), nuclear (Nu) and cytoskeletal (Ck) fractions. Scale bar: 20μm. (TIF) [file pone.0120781.s003.tif]

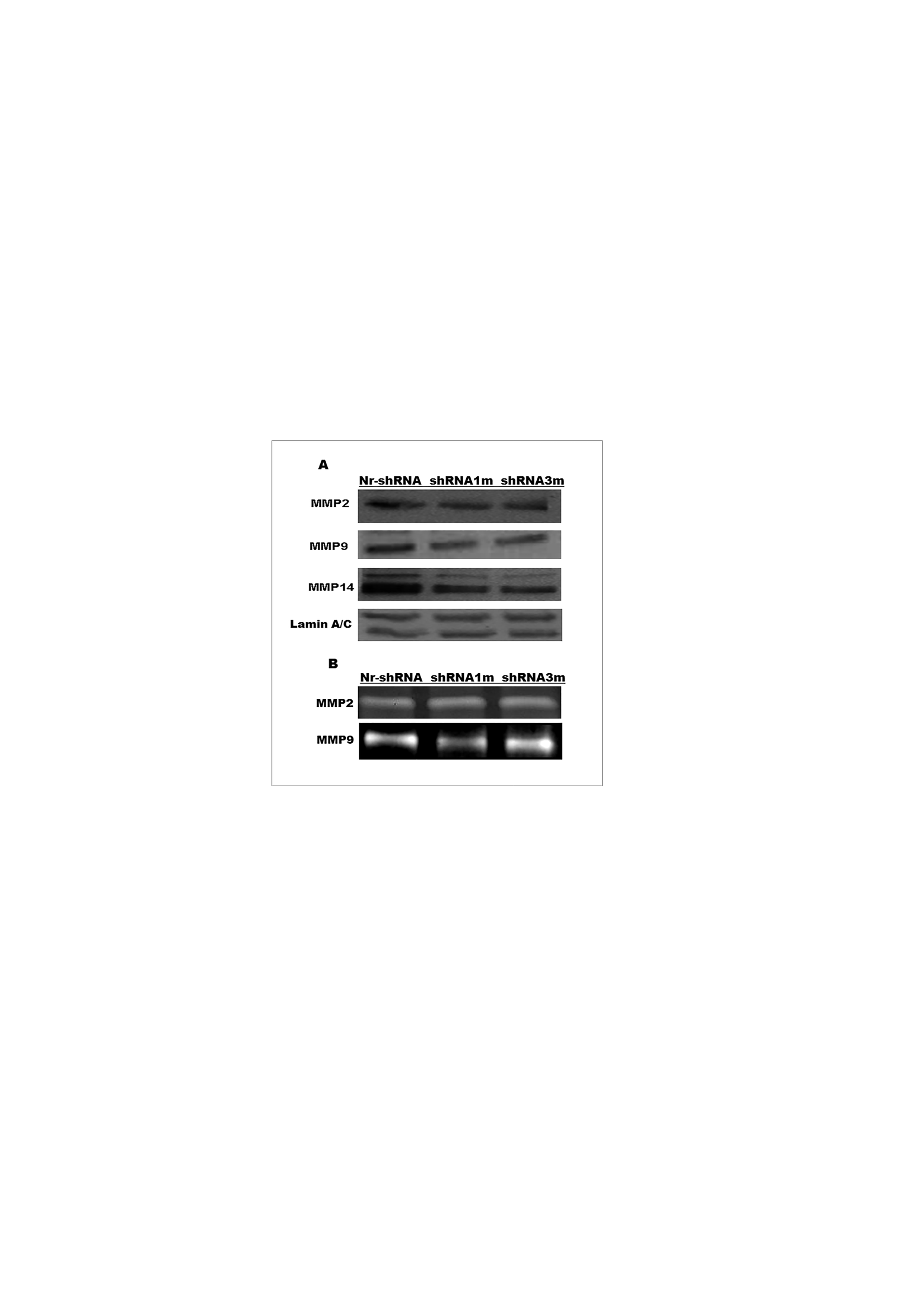

Supplement: S4 Fig — (A) Western blot showing expression of MMP2, 9 and 14. 72 h after transfection with shRNA plasmids (Nr-shRNA, shRNA 1m, shRNA 3m): 20 μg of protein from total lysates of cells were analyzed. (B), Representative zymogram of secreted MMP2 and MMP9. Control and depleted cells were serum-starved during 24 h, then secreted MMPs were quantified in culture supernatants by zymography. Spectrin depletion does not induce significant effects on metalloproteinases secretions. (TIF) [file pone.0120781.s004.tif]

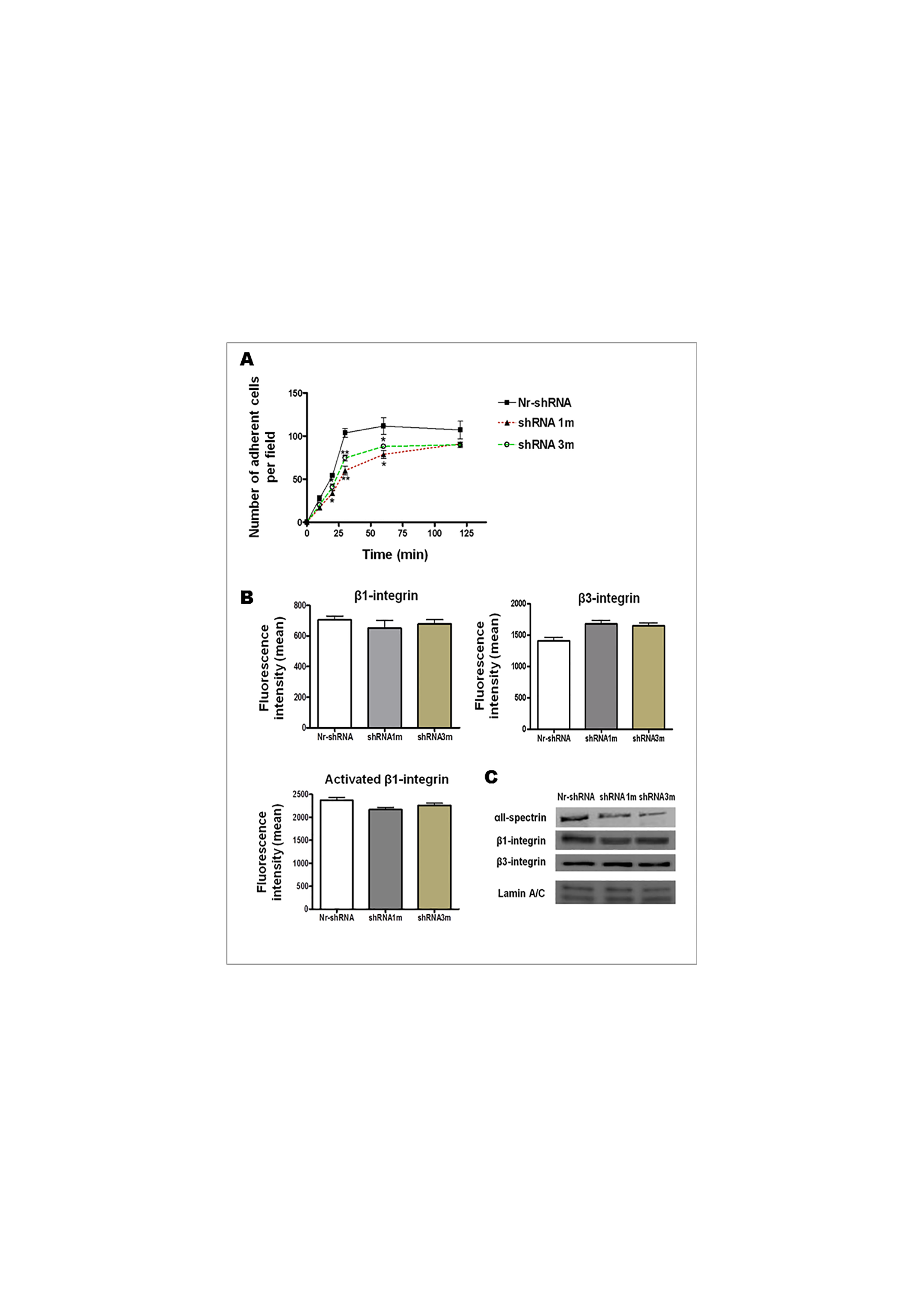

Supplement: S5 Fig — (A) SrcY527F-MEF cells were transfected for 96 h with shRNAs (Nr-shRNA, shRNA1m and 3m) and then seeded (100.000 cells) on plastic or vitronectin coated surface. At 10, 20, 30, 60 and 120 min, cells were gently washed and fixed, and the remaining cells corresponding to adherent cells were evaluated. (B) SrcY527F-MEF cells were transfected for 72 hr with irrelevant shRNA (Nr-shRNA) or αII-spectrin shRNAs (shRNA1m and 3m), and cell surface expression of β1-integrin, β3-integrin and an activated form of β1-integrin was analyzed by flow cytometry. αII-Spectrin silencing does not change significantly cell surface expression and activity of these integrins. (C) SrcY527F-MEF cells were transfected for 72 hr with irrelevant shRNA (Nr-shRNA) or αII-spectrin shRNAs (shRNA1m and 3m), and total expression of β1-integrin and β3-integrin was determined by western immunoblotting. αII-Spectrin silencing does not change significantly the expression of these integrins. (TIF) [file pone.0120781.s005.tif]
